# Detection of methylation in promoter sequences by melting curve analysis-based semiquantitative real time PCR

**DOI:** 10.1186/1471-2407-8-61

**Published:** 2008-02-25

**Authors:** Aiala Lorente, Wolf Mueller, Edurne Urdangarín, Paula Lázcoz, Andreas von Deimling, Javier S Castresana

**Affiliations:** 1 Brain Tumor Biology Unit, University of Navarra, Pamplona, Spain; 2 Department of Neuropathology, Institute of Pathology, Ruprecht-Karls-University, Heidelberg, Germany; 3 Department of Health Sciences, Public University of Navarra, Pamplona, Spain; 4 Clinical Cooperation Unit, Neuropathology, German Cancer Research Center (DKFZ), Heidelberg, Germany

## Abstract

**Background:**

We present two melting curve analysis (MCA)-based semiquantitative real time PCR techniques to detect the promoter methylation status of genes. The first, MCA-MSP, follows the same principle as standard MSP but it is performed in a real time thermalcycler with results being visualized in a melting curve. The second, MCA-Meth, uses a single pair of primers designed with no CpGs in its sequence. These primers amplify both unmethylated and methylated sequences. In clinical applications the MSP technique has revolutionized methylation detection by simplifying the analysis to a PCR-based protocol. MCA-analysis based techniques may be able to further improve and simplify methylation analyses by reducing starting DNA amounts, by introducing an all-in-one tube reaction and by eliminating a final gel stage for visualization of the result. The current study aimed at investigating the feasibility of both MCA-MSP and MCA-Meth in the analysis of promoter methylation, and at defining potential advantages and shortcomings in comparison to currently implemented techniques, *i.e. *bisulfite sequencing and standard MSP.

**Methods:**

The promoters of the *RASSF1A *(3p21.3), *BLU *(3p21.3) and *MGMT *(10q26) genes were analyzed by MCA-MSP and MCA-Meth in 13 astrocytoma samples, 6 high grade glioma cell lines and 4 neuroblastoma cell lines. The data were compared with standard MSP and validated by bisulfite sequencing.

**Results:**

Both, MCA-MSP and MCA-Meth, successfully determined promoter methylation. MCA-MSP provided information similar to standard MSP analyses. However the analysis was possible in a single tube and avoided the gel stage. MCA-Meth proved to be useful in samples with intermediate methylation status, reflected by a melting curve position shift in dependence on methylation extent.

**Conclusion:**

We propose MCA-MSP and MCA-Meth as alternative or supplementary techniques to MSP or bisulfite sequencing.

## Background

DNA methylation in promoter regions has a regulatory effect on gene transcription. The conversion by covalent binding of a methyl residue from cytosine to 5-methylcytosine (m^5^C) at CpG dinucleotides occurs in both, prokaryotic and eukaryotic genomes and represents the most abundant methylated base in the genome of vertebrates. Areas of high CpG dinucleotide density, so called "CpG islands", are spread throughout the genome and usually map to gene promoter regions. Almost half of the genes in our genome have such CpG-rich promoter regions [1]. Methylation of CpG islands is associated with histone deacetylation and transcriptional silencing [2] and it is essential for normal embryonic development, genomic imprinting and X-chromosome inactivation. It also plays a role in cancer, as tumor suppressor gene promoter methylation leads to their inactivation.

In standard PCR and cloning procedures, information about m^5^C and other covalent base modifications in genomic DNA is lost. Therefore, PCR methods for detecting and mapping m^5^C in specific genes rely on treatment of genomic DNA with methylation-sensitive restriction endonucleases or sodium bisulfite treatment before amplification. A specific target sequence can subsequently be amplified with primers specific for bisulfite-converted DNA and examined for its m^5^C content. The gold standard among bisulfite methods is genomic sequencing, that provides a positive display of m^5^C at specific CpG sites in virtually any stretch of DNA [3]. More simple methods using bisulfite-converted DNA as template include methylation-specific PCR (MSP) [4], methylation-sensitive single nucleotide primer extension [5] and procedures based on the use of restriction endonucleases [6,7]. MSP is designed to specifically amplify either methylated or unmethylated DNA by using primers that differentiate the bisulfite-converted methylated sequence from the unmethylated sequence [4]. Another method for analyzing promoter methylation status is the combined bisulfite restriction analysis (COBRA) [7]. The degree of methylation is determined by restriction enzyme digestion on CG-containing restriction sites, whereas methylated cytosine residues are protected from bisulfite conversion and subsequent digestion. The above methods entail a two step procedure, an initial PCR amplification and subsequent product analysis, usually by gel electrophoresis. Furthermore, with the exception of genomic sequencing, these are limited to the analysis of one or a few CpG sites in each setting.

A specific real-time quantitative MSP method based on detection of a fluorescent signal produced proportionally during polymerase chain reaction (PCR) amplification was developed in the 1990s and allows the rapid and highly accurate analysis of methylation levels in tissue samples [8,9]. More recently, a quantitative real-time methylation assay, which utilized the fluorescence-based TaqMan technology was developed [10]. This technique, called MethyLight, provides a sensitive and quantitative assay of methylated DNA. However, TaqMan technology requires the design of a fluorogenic probe, possibly a new set of primers and is relatively expensive.

Of note, high-throughput methodologies have been developed allowing quantitative methylation analyses targeting individual CpG dinucleotide residues, *i.e. *pyro-sequencing and matrix-assisted laser desorption/ionization time-of-flight mass spectrometry (MALDI TOF MS) [11,12]. However, both demand expensive hardware, which may not be easily accessible for many institutions, and either need time-consuming and sensitive samples preparation (MALDI-TOF) or are still limited to a rather short target sequence not suitable for screen purposes (pyrosequencing).

We describe 2 in-tube PCR assays for the detection of aberrant DNA methylation that use a thermal cycler integrated with a fluorometer and exploit differences in melting temperature (Tm) between methylated and unmethylated DNA after bisulfite treatment. Both real-time PCR techniques use the SYBR Green dye, which binds to double-stranded DNA. Bisulfite modified DNA is amplified and a melting curve of the PCR product is performed. After bisulfite conversion, the methylated sequence conserves the CG-dinucleotide residues, while these convert to TG dinucleotides in the unmethylated sequence, thereby reducing the melting temperature of the DNA fragment. Sequences with a high CG- to TG-conversion reflect unmethylated DNA. During melting curve analysis these will therefore reveal melting temperatures with peaks lower than those of CG-rich, methylated DNA sequences.

The first assay we describe is the Melting Curve Analysis-Methylation Specific PCR (MCA-MSP), that uses MSP primers for the amplification of bisulfite modified DNA. Like standard MSP it implements two sets of primers, one set specific for unmethylated and another set specific for methylated DNA. The reactions are carried out in two separate tubes.

Our second assay, the Melting Curve Analysis-Methylation assay (MCA-Meth), uses a single set of primers that amplifies both the methylated and the unmethylated DNA, as the primer-target annealing sequences, just like those of bisulfite sequencing primers, do not contain any CpG dinucleotides. The methylation status of the sample will be assigned according to the pattern of peaks in the melting curve.

We selected promoters of three tumor suppressor genes for our study: *RASSF1A *(3p21.3, a Ras oncogene associated protein that promotes cell cycle arrest in prometaphase), *BLU *(3p21.3, a DNA binding protein that regulates the entry of the cell into the cell cycle) and *MGMT *(10q26.1, a DNA repair gene that eliminates alkyl groups from the O^6 ^position of guanine). Many studies have been performed on the promoters of these genes demonstrating that the methylation status is relevant for tumor biology [13-19]. We have studied the methylation status and gene expression of these three tumor suppressor genes in 4 neuroblastoma cell lines, 6 astrocytoma cell lines and 13 primary astrocytic tumor samples.

## Methods

### Samples and cell lines

Thirteen primary astrocytic tumors and 10 cell lines (6 astrocytoma cell lines: A172, GOS-3, MOG-G-CCM, T98G, U87MG, U118; and 4 neuroblastoma cell lines: MC-IXC, SIMA, IMR32, SK-N-MC) were used for the study. Human samples were frozen at -80°C immediately after surgery. Cell lines were provided by the American Type Culture Collection (MC-IXC), the European Collection of Cell Culture (A172, MOG-G-CCM, T98G, and U87MG), the Deutsche Sammlung von Mikroorganismen und Zellkulturen (IMR-32, SIMA, SK-N-MC, GOS-3) and the American Type Cell Collection (U118).

MC-IXC, IMR32, SIMA and SK-N-MC cell lines were grown with Dubelcco's modified Eagle's medium (DMEM+ L-Glutamax), supplemented with 10% fetal bovine serum (FBS), 1% penicillin/streptomycin and 0.1% anfotericine B, at 37°C with 5% CO_2_. GOS-3, T98G, MOG-G-CCM, A172, U87MG and U118 cell lines were cultured with RPMI+L-Glutamax medium, supplemented with 10% fetal bovine serum (FBS), 1% penicillin/streptomycin and 0.1% anfotericine B, at 37°C with 5% CO_2_.

### DNA extraction and bisulfite treatment

DNA from the cell lines was purified by the Wizard^® ^Genomic DNA Purification Kit (Promega) according to manufacturer's instruction. 1 μg genomic DNA was bisulfite modified by the CpGenome™ DNA Modification Kit (Chemicon^® ^International), following manufacturer's protocol. Modified DNA was purified, then eluted in 1 mM TE pH 8, and used immediately or stored at -80°C for up to six months. Blood genomic DNA and in vitro methylated DNA (Genome™ Universal Methylated DNA, Chemicon^® ^International), were used as negative and positive controls, respectively, for the methylation status of DNA.

### MSP

The methylation specific PCRs were carried out with 60 ng of bisulfite modified DNA in a total volume of 25 μl, which contained 2.5 μl 10× reaction buffer, 2–2.5 mM MgCl_2_, 0.2 mM of each dNTP, 10 pmol forward and reverse primers, 5% DMSO and one unit of AmpliTaq Gold™ polymerase (Roche), in a T3 thermocycler of Biometra^®^.

Oligonucleotides for *RASSF1A *and *MGMT *(Table 1) were designed using the MethPrimer software [20]. Primers for *BLU *(Table 1) were described previously [13]. PCR reactions were denatured at 94°C for 10 min, followed by 36–38 cycles of 45 s at 94°C, 30–45 s at the corresponding annealing temperature for each gene (Table 1), 30–45 s at 72°C, followed by a final extension step at 72°C for 10 min. PCR products were visualized in a 2% agarose gel stained with ethidium bromide at a final concentration of 0.1 μg/ml.

**Table 1:** List of oligonucleotides used for MSP, MCA-Meth and bisulfite sequencing of *RASSF1A*, *MGMT *and *BLU *genes.

	MSP and MCA-MSP	MCA-Meth	bisulfite sequencing
*RASSF1A*	UF: 5'-GAGAGTGTGTTTAGTTTTGTTTTT-3'	F: 5'-AGTTTTTGTATTTAGGTTTTTATTG-3'	F: 5'-ATAGTAAAGTTGGTTTTTAGAAATA-3'
	UR: 5'-CCCATACTTCACTAACTTTAAACAC-3'	R: 5'-AACTCAATAAACTCAAACTCCCC-3'	R: 5'-CAACTCAATAAACTCAAACTCCCCC-3'
	MF: 5'-GAGAGCGCTTTAGTTTCGTTTTC-3'		
	MR: 5'-ACCCGTACGTTCGCTAACTTTAAACG-3'		
*MGMT*	UF: 5'-GAGAGATTTGTGTTTTGGGTTTAGTG-3'	F: 5'-GGTTTGGGGGTTTTTGATTAG-3'	F1: 5'-GGTATTAGGAGGGGAGAGATT-3'
	UR: 5'-CCTTCAACCAATACAAACCAAACAA-3'	R: 5'-CCTTTTCCTATCACAAAAATAATCC-3'	R1: 5'-TATACCTTAATTTACCAAATAACCC-3'
	MF: 5'-ATTCGCGTTTCGGGTTTAGC-3'		F2: 5'-GTAAATTAAGGTATAGAGTTTTAGG-3'
	MR: 5'-CGACCGATACAAACCGAACG-3'		R2: 5'-AACTATCCCAACATATCC-3'
*BLU*	UF: 5'-TTTGTGGGTTATAGTTTGAGAAAGTG-3'	F: 5'-AAGGATTTGGAGTTTAGGAGAGATT-3'	F: 5'-AAGGATTTGGAGTTTAGGAGAGATT-3'
	UR: 5'-AACAAATTAACCACACCTACAC-3'	R: 5'-CCAAAATCTAAAACAAAACAATTAC-3'	R: 5'-CCAAAATCTAAAACAAAACAATTAC-3'
	MF: 5'-TTCGTGGGTTATAGTTCGAGAAAGCG-3'		
	MR: 5'-AACGAATTAACCGCGCCTACGC-3'		

Annealing temperatures for every set of primers:

MSP: RASSF1A UF and UR: 62°C; RASSF1A MF and MR:58°C; MGMT UF and UR: 62°C; MGMT MF and UR: 62°C; BLU UF and UR: 60°C; and BLU MF and MR: 66°C.

MCA-Meth: RASSF1A F and R: 58°C; BLU F and R: 56°C; and MGMT F and R: 61°C.

Bisulfite sequencing: RASSF1A F and R: 61°C; MGMT F1 and R1: 58°C; MGMT F2 and R2: 58°C; and BLU F and R: 56°C.

### MCA-MSP

MCA-MSP (Melting Curve Analysis-Methylation Specific PCR) was carried out with 3–6 ng of bisulfite modified DNA in a total volume of 25 μl, containing 12.5 μl of 2 × IQ™ SYBR Green Supermix (Bio-Rad) and 2.5 pmol of primers (either the primers for the methylated or the unmethylated sequence). The primers for *RASSF1A*, *MGMT *and *BLU *genes are the ones described for MSP (see Table 1).

The reactions were heated at 94°C for 10 min, followed by 45 cycles of 30 s at 94°C, 30 s at the corresponding annealing temperatures (Table 1), 30 s at 72°C, and 30 s at 76–77°C. We modified the MSP protocol by addition of an extra incubation step at 76–77°C in each cycle, to overcome the primer dimer artifact, as described previously [21]. After gene amplification, the melting curve analysis was performed as follows: from 70°C to 90°C, the temperature was increased by 0.5°C every 30 s. Both amplification and melting curve were carried out in an IQ5 Multicolor Real-Time PCR Detection System (Bio-Rad).

### MCA-Meth

MCA-Meth (Melting Curve Analysis-Methylation assay) was carried out with 3–6 ng of bisulfite modified DNA in a total volume of 25 μl, containing 12.5 μl of 2× IQ™ SYBR Green Supermix (Bio-Rad) and 2.5 pmol of primers.

The primers for *RASSF1A*, *MGMT *and *BLU *genes (Table 1) were designed using the MethPrimer software [20]. These primers do not contain any CpG in their sequence. Therefore they will amplify the target DNA regardless of its methylation status. The reactions were heated at 94°C for 10 min, followed by 40–45 cycles of 30 s at 94°C, 30 s at the corresponding annealing temperature (Table 1), and 30 s at 72°C. For the *MGMT *gene, an extra step of 30 s at 76°C was added in each cycle, to avoid the signal of primer dimers. The melting curve was performed following the same protocol as for the MCA-MSP assay. Both amplification and melting curve were carried out in an IQ5 Multicolor Real-Time PCR Detection System (Bio-Rad).

### Bisulfite sequencing

Initially, 12 ng of bisulfite treated DNA of a target promoter region were amplified in a PCR reaction with a total volume of 20 μl, containing 10 μl of 2× PCR Mastermix-Y (PEQLAB) and 5 pmol of bisulfite sequencing primers. The oligonucleotides were designed using the MethPrimer software. The reactions were denatured at 95°C in a first PCR step for 5 min, then 38 cycles of 30 s at 95°C, 1 min at the corresponding annealing temperature for each gene (Table 1), and 2 min at 72°C, followed by a final extension step at 72°C for 10 min, in a T Gradient thermocycler (Biometra).

Secondly, the PCR product was plasmid incorporated using the One Shot *E. coli *cells and the TOPO TA Cloning Kit PCR^®^2.1-TOPO Vector (Invitrogen). Cells were then plated and grown overnight on LB plates containing X-Gal and 50 mg/ml ampicillin and 50 mg/ml kanamycin. In order to validate the colonies with the right insert, colony PCR was then performed on 8 white colonies, with 10 pmol of M13 primers and 10 μl of 2× PCR Mastermix-Y (PEQLAB) in a total volume of 20 μl. The reactions were heated at 94°C in a first PCR step for 5 min; 33 cycles of 30 s at 94°C, 1 min at 55°C, and 10 s at 72°C; followed by an extension step of 10 min at 72°C.

PCR products harboring the insert were digested with EXOSAP-IT^® ^exonuclease (USB Corp.) and the sequencing PCR was performed with 1 μl Big Dye^® ^Terminator v1.1 cycle sequencing PR-100, 1.5 μl Big Dye^® ^Terminator v1.1, v3.1 5× sequencing buffer (Applied Biosystems), 5 pmol of M13 reverse primer and 1 μl of digested DNA in a total volume of 10 μl. The reaction mixture was denatured at 93°C for 5 min, followed by 25 cycles of 10 s at 96°C, 15 s at 57°C and 4 min at 60°C. This sequencing PCR product was cleaned in Sephadex^® ^G-50 (Sigma-Aldrich) columns and then sequenced in an ABI PRISM 377 DNA Sequencer (Applied Biosystems). The results were analysed using the Sequencher Software version 4.2.

## Results

### Optimizing the real-time PCR

#### Diluting the template DNA

In order to optimize the conditions for the real time PCRs, we tried to minimize as much as possible the amount of DNA used in the PCR reactions. For standard MSP we usually take 60 ng of bisulfite converted DNA. We tried 1:5 (12 ng), 1:10 (6 ng), 1:20 (3 ng), 1:30 (2 ng), 1:40 (1.5 ng) and 1:50 (1.2 ng) dilutions of the bisulfite modified DNA for our real-time PCR experiments. As the amount of DNA was decreased, the amplification started later, giving higher C_T _(threshold cycle) values: C_T _= 34.7 for 1:5 dilution, C_T _= 37.6 for 1:10 dilution, C_T _= 38.7 for 1:20 dilution, C_T _= 39.7 for 1:30 dilution and C_T _= 40.5 for 1:40 dilution. We did not obtain any results with the 1:50 dilution. We also observed that as the amount of DNA decreased, the reproducibility of the reaction was lower, giving a higher inter-tube and inter-assay variation, mainly in the 1:30 (2 ng) and 1:40 (1.5 ng) dilutions (data not shown). Thus, we recommend using the 1:10 (6 ng) or 1:20 (3 ng) dilution to obtain a good balance between DNA economy and an assay of good quality.

#### Overcoming primer dimers

The SYBR-Green dye binds to double stranded DNA. This holds true for the specific product but also for primer dimers, if they are formed during the PCR reaction. To overcome this problem we included an extra incubation step of 76–77°C for 30 s after the 72°C extension step [21] in the PCRs in which primer dimers gave a strong signal. The extra incubation step dissociates the double-stranded primer dimers into single-stranded DNA while leaving the PCR products intact. Thus the fluorescence signal obtained at this higher temperature is only due to the specific PCR products. The temperature of this extra step has to be higher than the melting temperature of the primer dimers and lower than the melting temperature of the specific products, and can vary between different PCRs.

The extra step was added in the two PCRs performed for every gene studied by MCA-MSP, as primer dimers were generated in all these cases. In contrast, it was only necessary for *MGMT *amplification by MCA-Meth (Table 2).

**Table 2:** C_T _values obtained after MGMT amplification of no methylated DNA (from blood), in vitro methylated DNA (IMD) and no template control (H_2_O) by MCA-Meth analysis, with and without the adition of the extra incubation step. C_T _values get reduced (primer dimers do not get amplified) when using the incubation step. No amplification at all is produced in the no template control (H_2_O), meaning that the C_T _value of 36.8 corresponded to primer dimer amplification.

	Blood	IMD	H_2_O
C_T _without step	35.5	38.6	36.8
C_T _with step	34.2	38.3	-

### Comparison of the MSP, MCA-MSP and MCA-Meth techniques

**Figure 1:**
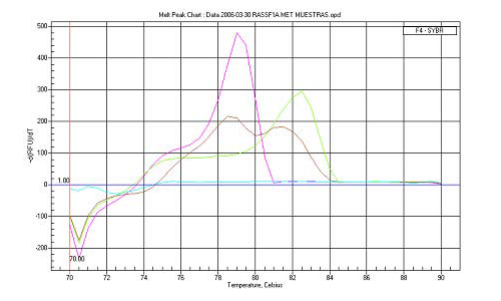
**MCA-Meth analysis in *RASSF1A *gene for the astrocytoma sample 33**. The graph shows the unmethylated control peak (blood DNA) in red, the methylated control peak in green, the no template control in blue, and the sample with both the unmethylated and the methylated peaks, in brown. This result suggests a mixture of methylated and unmethylated DNA in this sample. It might be due to the contamination of the tumor sample with normal adjacent tissue.

Promoter hypermethylation of *RASSF1A*, *BLU *and *MGMT *was assessed by MCA-MSP and MCA-Meth (Figure 1), and compared to conventional MSP. The three techniques were significant for *RASSF1A *promoter hypermethylation in all tumor samples and all glioma cell lines.

**Figure 2:**
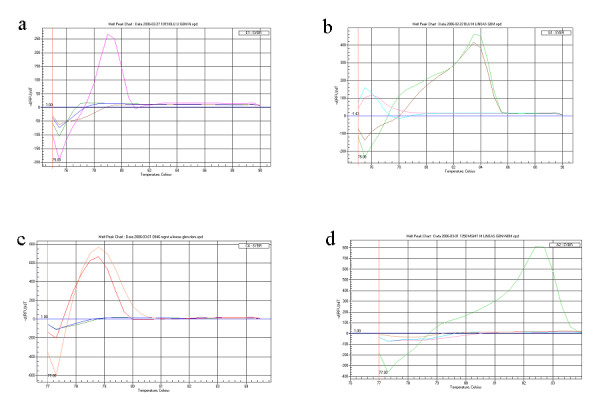
**MCA-MSP analysis in *BLU *and *MGMT *genes for the A172 and SIMA cell lines**: The graphs show the unmethylated control peak (blood DNA) in red, the methylated control peak in green, the no template control in blue and the cell line peak, in brown. **a) **PCR for the unmethylated sequence, *BLU *gene, A172 cell line: only the blood DNA gives a peak, thus the cell line contains no unmethylated DNA. **b) **PCR for the methylated sequence, *BLU *gene, A172 cell line: the methylated DNA and the cell line give peak, thus the A172 cell line is methylated for the BLU gene. **c) **PCR for the unmethylated sequence, *MGMT *gene, SIMA cell line: the blood DNA and cell lines give peak, thus the cell line is unmethylated. **d) **PCR for the methylated sequence, *MGMT *gene, SIMA cell line: only the methylated DNA gives a peak, thus the cell line is not methylated.

MSP revealed *BLU *promoter hypermethylation in cell lines GOS-3, A172, MOG-G-CCM, SIMA and IMR-32. U87MG, T98G, U118, SK-N-MC and MC-IXC were unmethylated. Similar results were obtained by MCA-MSP or MCA-Meth in all cell lines except for IMR32, which appeared unmethylated in the MCA-MSP and MCA-Meth analyses (Figure 2).

We received matching results in all three techniques for 10 out of 13 tumor samples for the *BLU *gene promoter. Two tumors were methylated and eight were unmethylated. All three remaining tumors appeared methylated by MSP, while two were unmethylated amd one was methylated by MCA-MSP and MCA-Meth (Tables 3 and 4).

**Table 3:** *BLU*, *RASSF1A *and *MGMT *genes hypermethylation in astrocytoma and neuroblastoma cell lines.

	*BLU*					*RASSF1A*					*MGMT*				
	MSP	MCA-MSP	MCA-Meth		Seq	MSP	MCA-MSP	MCA-Meth		Seq	MSP	MCA-MSP	MCA-Meth		Seq
			
U87	U		U	0%		U+M		U+M	36%		M		UM		UM
GOS 3	M	M	M	100%		M	M	M	100%		U+M	U+M	U+M	78%	
A 172	M	M	M	100%	M	M	U+M	M	100%		M	M	UM		
MOG	U+M	M	U+M	53%		M	UM	UM			U+M	UM	UM		
T98G	U	U	U	0%		M	M	M	67%	M	M	UM	M	61%	
U118	U		U	0%	U	M		M	100%		M		M	98%	
SIMA	U+M	M	M	76%		M	M	M	100%		U	U	U	0%	
IMR 32	U+M	U	U	0%		M	M	M	98%		U+M	UM	M	86%	
SK-N-MC	U	U	U	0%		M	M	M	100%		U+M	U	UM		
MC-IXC	U	U	U	0%		M	M	M	100%		U	U	U	0%	

U, unmethylated peak or band; M, methylated peak or band; U+M, sample producing both methylated and unmethylated peaks or bands; MU, sample producing only one peak at an intermediate temperature; blank spaces: not determined analyses. The percentage of fully methylated DNA molecules present in the samples (semiquantitative methylation study) is shown for the MCA-Meth in the M and U+M cases. On the contrary, no percentage appears in the the UM cases, as the UM corresponds to partially methylated DNA molecules, that can not be semiquantitatively studied according to the curve obtained with different proportions of fully methylated DNA.

**Table 4:** *BLU*, *RASSF1A *and *MGMT *genes hypermethylation in astrocytoma tumor samples.

		*BLU*				*RASSF1A*					*MGMT*				
Sample		MSP	MCA-MSP	MCA-Meth		MSP	MCA-MSP	MCA-Meth		Seq	MSP	MCA-MSP	MCA-Meth		Seq
			
7	A III	U		U	0%	U+M		U+M	<5%	U	U+M		U+M	<5%	
8	A III-IV	U	U	U	0%	U+M	U+M	U+M	<5%		U+M	U	U+M	<5%	
12	GBM	U	U	U	0%	U+M	M	U+M	<5%		U	U	U	14%	
13	GBM	U+M	U+M	U	22%	U+M	M	U+M	20%		U	U	U	0%	
26	GBM	U+M	U	U	0%	U+M	M	U+M	<5%		U	U	U	0%	U
28	GBM	U+M	U	U+M	<5%	U+M	M	U+M	12%		M	U+M	M	100%	M
30	GBM	U+M	U+M	U+M	65%	U+M	U+M	U+M	72%	M	U+M	U+M	UM		
33	A III	U+M	U+M	U+M	57%	U+M	U+M	U+M	46%		U+M	U+M	UM		UM
45	GBM	U	U	U	0%	U+M	U+M	U+M	<5%		U+M	U	U	0%	
46	GBM	U	U	U	0%	U+M	U+M	U+M	<5%		U+M	U	U+M	<5%	
47	A III-IV	U	U	U	0%	U+M	U+M	U+M	22%		U+M	U	U+M	<5%	
49	GBM	U	U	U	0%	U+M	U+M	U+M	<5%		U	U	U	0%	
51	GBM	U	U	U	16%	U+M	U+M	U+M	<5%		U+M	U	UM		

U, unmethylated peak or band; M, methylated peak or band; U+M, sample producing both methylated and unmethylated peaks or bands; MU, sample producing only one peak at an intermediate temperature; blank spaces: not determined analyses. The percentage of fully methylated DNA molecules present in the samples (semiquantitative methylation study) is shown for the MCA-Meth in the M and U+M cases. On the contrary, no percentage appears in the the UM cases, as the UM corresponds to partially methylated DNA molecules, that can not be semiquantitatively studied according to the curve obtained with different proportions of fully methylated DNA.

**Figure 3:**
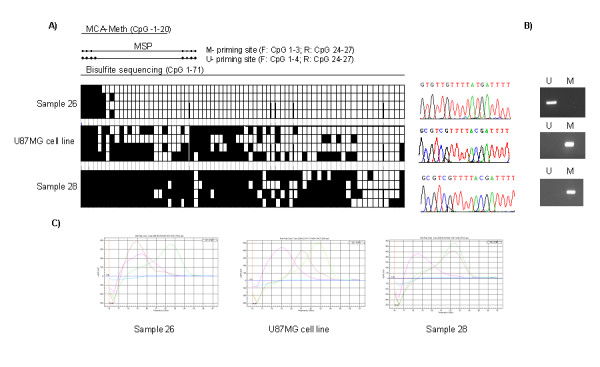
**Hypermethylation analysis by bisulfite sequencing, MSP and MCA-Meth of *MGMT *gene in astrocytoma samples 26, 28 and in the U87MG cell line**. **a) **Bisulfite sequencing results of the *MGMT *gene in astrocytoma samples 26 and 28 and in the U87MG cell line. Black squares indicate methylated CpG dinucleotides and white squares indicate unmethylated CpG dinucleotides in promoter sequences. Above the diagrams, the area of the promoter analyzed by MSP, MCA-Meth and bisulfite sequencing is indicated. The 5'- and 3'-priming sites of M- and U-primers are indicated as small black dots representing the CpG sites incorporated in the primer sequence. The position of CpGs in relation to the CpG-island as well as the number of CpGs analyzed are indicated in the brackets behind the respective techniques. On the right, the diagrams of peaks obtained with the sequencing software: it can be observed how after the bisulfite treatment, the cytosines remain unchanged in the methylated sample, while there is a shift to thymine in the unmethylated sample. **b) **MSP results of *MGMT *in astrocytoma samples 26, 28 and in the U87MG cell line. U: PCR with primers specific for the unmethylated sequence; M: PCR with primers specific for the methylated sequence. **c) **MCA-Meth results of *MGMT *in astrocytoma samples 26, 28 and in the U87MG cell line. The graphs show the unmethylated control peak (blood DNA) in red, the methylated control peak in green, the no template control in blue and the sample or cell line peak, in brown. It can be observed how in the case of the U87MG cell line, the amount of methylated cytosines in the area of the promoter analyzed by bisulfite sequencing is intermediate between the sample 26 (unmethylated) and sample 28 (fully methylated). Of note, MCA-Meth appreciates this inhomogeneous sequence methylation pattern of U87MG with a melting curve peak in an intermediate position as compared to the unmethylated (26) and the methylated sample (28).

GOS-3, A172, MOG-G-CCM, T98G, U87MG, U118 and IMR-32 cell lines showed *MGMT *promoter hypermethylation by all three techniques, while SIMA and MC-IXC harbored an unmethylated promoter. Cell lines SK-N-MC and MC-IXC produced ambiguous results. For SK-N-MC *MGMT *promoter hypermethylation was detected by both MCA-MSP and MCA-Meth, whereas conventional MSP was significant for an unmethylated *MGMT *promoter. For MC-IXC MCA-Meth analysis showed evidence of promoter hypermethylation but MSP and MCA-MSP were in favor of an unmethylated promoter. In 8 of 13 tumor samples all three techniques produced homogeneous results (4 unmethylated and 4 methylated *MGMT *promoters). Conflicting results were seen in the other 5 tumors. One of these samples showed hypermethylation by MSP but neither by MCA-MSP nor MCA-Meth. The remaining 4 samples were positive for a hypermethylated promoter by MSP and MCA-Meth, but revealed no evidence for promoter methylation by MCA-MSP (Tables 3 and 4, Figure 3).

### Validation of the power of the presented techniques of promoter hypermethylation with bisulfite sequencing

In most of the samples analyzed, the results obtained with the 3 techniques matched. However, ambigiuous results were obtained for some samples. This might be due to a difference in the sensitivity of the techniques to detect the hypermethylation or to the fact that the primers used in the different assays do not exactly target the same CpGs in the promoter region. To clarify this question, we performed bisulfite sequencing of the area of the promoter covered by the two types of primers used for the different techniques in each gene.

Also, we observed that in the MCA-MSP or MCA-Meth techniques some samples gave a peak in the melting curve at an intermediate temperature between the peaks corresponding to the fully methylated or unmethylated DNA. These peaks suggest an intermediate amount of methylated cytosines in the samples. Bisulfite sequencing was performed to verify our hypothesis.

Two cell lines were sequenced in the promoter of the *BLU *gene: U118 and A172. Bisulfite sequencing revealed that U118 was unmethylated and A172 was methylated. These results were consistent with the results obtained with MSP and MCA-Meth techniques (Table 3).

Two astrocytomas (samples 7 and 30) and the cell line T98G were sequenced in the promoter of the *RASSF1A *gene: sample 30 and the T98G cell line were strongly methylated while sample 7 presented a lower degree of methylation (3 to 16 of the 32 CpGs analyzed were methylated in the different clones). Sample 30 and the T98G cell line appeared methylated by all the other techniques as well. However, sample 7 was methylated by MSP and MCA-Meth, the latter in a very low proportion (Tables 3 and 4).

Two other astrocytomas (samples 26 and 28) and the U87MG cell line were sequenced in the promoter of the *MGMT *gene (Figure 3): sample 26 was unmethylated and sample 28 was strongly methylated. These results are consistent with the results obtained with the other 3 techniques (Table 4). U87MG cell line was "half" methylated by bisulfite sequencing, corresponding with a peak in an intermediate position in the MCA-Meth. MSP results show that this cell line is methylated (Table 3). Thus, the MCA-Meth and MCA-MSP techniques can detect the half-methylated status of the promoter and differentiate it from a fully methylated promoter, whereas MSP can not.

### Semi-quantification of the methylation status by MCA-Meth

**Figure 4:**
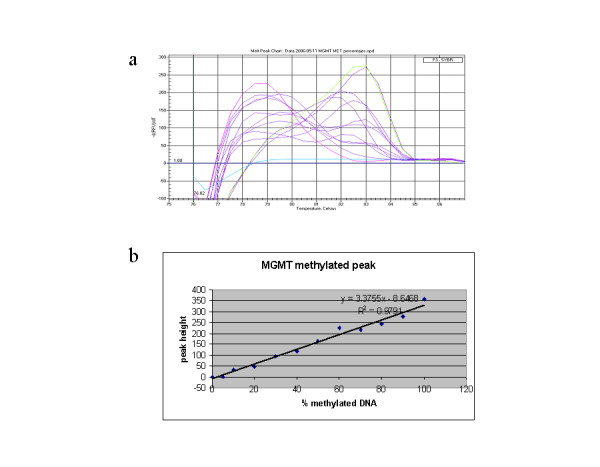
**Determination of the percentage of fully methylated *MGMT *by MCA-Meth**. **a) **The graph shows the melting peaks for the different percentages of methylated and unmethylated DNA. The increase and the decrease in the height of the specific peaks in the different percentages can be observed. Red: 0% methylated DNA. Green: 100% methylated DNA. Purple: 5%, 10%, 20%, 30%, 40%, 50%, 60%, 70%, 80% and 90% methylated DNA. **b) **Graph representing the linear relationship between the % of methylated DNA and the height of the methylated peak in the melting curve of the MGMT gene. As the % of methylated DNA increases (0%, 5%, 10%, 20%, 30%, 40%, 50%, 60%, 70%, 80%, 90% and 100%) the peak appears higher. Measuring the height of the peak of each sample, and interpolating the data in the graph, we can obtain an approximate % of fully methylated DNA present in each sample.

We mixed unmethylated and methylated DNA at different percentages (0%, 5%, 10%, 20%, 30%, 40%, 50%, 60%, 70%, 80%, 90%, and 100%). Then we performed the MCA-Meth analysis for the three genes (*RASSF1A*, *BLU *and *MGMT*) in triplicate and measured the height of the peaks obtained and calculated the average height for each triplicate. As expected, the 0% gave the unmethylated peak only and 100% only the methylated peak, while the intermediate percentages gave the two peaks, increasing the height of the unmethylated peak proportionally to the amount of unmethylated DNA and increasing the height of the methylated peak proportionally to the amount of methylated DNA (Figure 4). Then, we measured the triplicates of the peaks of the tumor samples and cell lines and interpolated those values into the graphs obtained with the percentages above (Figure 4), to obtain a semiquantitative idea of the amount of methylated-unmethylated DNA (Table 3, Table 4).

## Discussion

Several methodologies have been developed to assess promoter methylation. We present here two techniques that exploit the differences in melting temperature (Tm) between methylated and unmethylated alleles after bisulfite treatment and allow for the distinction of alleles harboring different percentages of methylated CpG dinucleotides. After bisulfite treatment, a methylated sequence conserves the CG pairs while an unmethylated sequence presents changes to TG pairs. This change in CG content results in a variation of the melting temperature of a particular DNA fragment. Based on the melting temperatures and melting curve shapes, it was possible to assign a methylation status to the tested samples.

Melting curves of methylated and unmethylated DNAs are tested in two separate reactions when using Melting Curve Analysis-Methylation Specific PCR (MCA-MSP), as in standard MSP. Thus, we simply look for the presence or absence of product amplification that will give a single melting curve peak (Figure 2). Therefore, MCA-MSP does not require a difference in the Tm between the methylated and unmethylated DNA. In theory, it may be possible to multiplex MCA-MSP assays by performing a single PCR reaction that includes both methylated and unmethylated specific primers. However, multiplexing assays would require a difference in the Tm between methylated and unmethylated peaks, and both methylated and unmethylated specific primers to anneal at the same temperature during the PCR.

The Melting Curve Analysis-Methylation assay (MCA-Meth) only uses one set of primers that do not include methylation susceptible CpGs on its sequence. This set of primers, similar to bisulfite sequencing primers, amplifies both the methylated and the unmethylated sequence. The difference will become evident in the melting curves, as the methylated product will have a higher melting temperature than the unmethylated product (Figure 1, Figure 3). However, it can be difficult to find a good set of primers with these characteristics for some genes.

The two techniques described here, both using the SYBR Green dye and Melting Curve Analysis, provide several advantages over standard MSP and other commonly used methylation screening methods. Experimental protocols are simple, can be carried out in high throughput formats (96 or 384-well microplates) allowing very little sample manipulation and do not need a gel phase for result documentation. Classical MSP necessitates 10–20 ng DNA, while successful MSP-Meth amplification is possible with as little as 6 ng DNA. This may be especially important when analyzing DNA derived from archival material. An additional advantage of the MCA-Meth over MCA-MSP is that the analysis can be done in a single all-in-one-tube-reaction, instead of two reactions usually required for MCA-MSP and MSP. Thereby, it is easier to handle, less prone to contamination by other samples, saves DNA for additional analyses on those samples and shortens analysis time.

An advantage of these real time based techniques is the possibility of quantifying the presence of methylated and unmethylated DNA. If primer dimer signals were avoided, and if there were no unspecific product amplification, the quantity of fluorescence detected should be proportional to the quantity of specific amplification product, giving the possibility of quantification. In MCA-MSP, quantification can be done by analyzing the amplification curve taking into account the C_T _(threshold cycle). Other authors performed and compared MCA-MSP and real-time quantitative PCR methylation using TaqMan probes. With the MCA-MSP protocol, the C_T _values at most concentrations were comparable to TaqMan, but the sensitivity was lower [21]. We hypothesized that the height of the peaks was proportional to the amount of product in MCA-Meth. To confirm this hypothesis, we performed a series of dilutions of fully methylated DNA and run MCA-Meth PCRs for three genes (*RASSF1A*, *BLU *and *MGMT*) in triplicate, measured the height of the peaks obtained and calculated the average height for each triplicate. We obtained a linear relationship between the percentage of fully methylated DNA and the height of the peak (Figure 4). We succeeded in detecting as little as 5% of methylated DNA, with 6 ng of starting DNA in the PCR. The sensitivity of the assay might be higher if the amount of template DNA in the reaction was increased.

Besides distinguishing methylated from unmethylated DNA, MCA-Meth provides the possibility to detect an intermediate status of methylation. We observed these cases as peaks in an intermediate position, when compared to the unmethylated and methylated control peaks (Figure 3, U87MG cell line). Temperature transition rates and concentration of salt and dye may have a significant impact on the width and absolute position of a PCR product's melting peak [22]. We observed a variation of 1°C for the unmethylated blood DNA peak and 1.5°C for the *in vitro *methylated DNA peak (negative and positive controls, respectively) as a normal variation between different experiments (data obtained from the comparison among 20–30 experiments for each gene, data not shown). To visualize and ensure the respective methylation status, both controls were bisulfite-sequenced prior to further experiments.

Both MCA-MSP and MCA-Meth have been proven here to reliably detect promoter methylation. However, the results obtained with these techniques versus standard MSP were not the same in some cases. These aberrant results may highlight advantages and shortcomings of both standard MSP and MSP-derived assays and MCA-Meth and may root in the different technical approaches of these assays. As mentioned above in standard MSP only the CpGs incorporated into the primer sequences decide on the methylation status of the gene promoter as a whole. Therefore MSP and MSP-derived methods depend on homogeneous methylation patterns at their respective priming sites. MCA-Meth, however, is independent of homogeneous methylation patterns as the priming sites are devoid of CpG-dinucleotide residues. Rather, melting curve peak position in relation to unmethylated and methylated DNA controls decides on the promoter methylation status. This independence of homogeneous priming site methylation patterns makes the analysis of inhomogeneously methylated DNA samples possible. The results may also better correlate with sequencing data, as the melting curve peak position shifts to a higher temperature the more CpGs are methylated within a given sequence. In cases with inhomogeneously methylated MSP priming sites MCA-Meth may be superior to standard MSP. Also, there is the possibility that sequence methylation in between the MSP-primers significantly impacts the extent of gene silencing and differs among samples with homogeneously methylated priming sites. This would lead to the overestimation of promoter hypermethylation and to false positive results unwanted in a diagnostic setting. Bisulfite sequencing easily closes that gap, as it reflects the CpG dinucleotide methylation status of the sequenced promoter region. Yet, it necessitates good quality DNA, as the sequenced fragment usually exceeds the size of a MSP product. Also, it calls for cloning the fragments into plasmids in order to secure clean sequencing results. For semiquantification, multiple clones have to be sequenced and all CpGs within the sequence have to be analyzed after sequencing, making bisulfite-sequencing a time- and labor-consuming technique, not suited for fast-track diagnostic testing. In these situations MCA-Meth may proof a useful alternative screening tool.

Standard MSP will, however, be superior to MCA-Meth in cases in which priming site methylation is homogeneously found and reliably predicts gene silencing. Again MSP priming site methylation may be homogeneous for these promoters, but methylation frequency may differ in the sequence between the primers. In those situations it is conceivable that MCA-Meth yields results that are hard to interpret as it does not detect the priming site methylation status but rather relies on the methylation pattern of the whole sequence analyzed.

Figure 3 shows the possible impact of inhomogeneous priming site methylation on MSP results as well as the influence of the extent of sequence methylation on MCA-Meth. Bisulfite sequencing of sample 26 revealed an almost completely unmethylated promoter except for partial methylation of the first five to six CpGs of the CpG-island (Figure 3). Even though these methylated CpG-residues were part of the 5'-priming site selected for MSP primers, MSP was significant for an unmethylated promoter, as well. The explanation for the seemingly paradoxical MSP result may lie in both the homogeneous 3'-priming site methylation pattern and an inhomogeneous 5'-priming site methylation coupled with differences in M- and U-primer design. As documented in bisulfite sequencing all four clones harbor a completely unmethylated 3'-priming site. Primer sequences of both M- and U-primers cover the same 4 CpG-dinucleotides of the CpG-island. As these CpGs (CpG 24–27) are unmethylated in all clones, 3'-mispriming seems impossible. The 5'-priming site is inhomogeneously methylated with either the first five or six CpGs methylated in bisulfite sequencing. We sequenced four clones only, but it seems conceivable that with an inhomogeneous methylation pattern on the 5'-priming site, there may be clones in which less than the first five CpGs are methylated. As the 3'-priming site already selects for the unmethylated allele, only a few copies of a less methylated 5'-priming site would suffice to yield an unmethylated promoter, explaining the MSP result. What is more as the 5'-priming site for the unmethylated variant of the *MGMT*-promoter exceeds the 5'-priming site for the methylated variant by one CpG-dinucleotide (Figure 3). The additional CpG dinucleotide lies on the 3'-end of the U-primer. Primer annealing at the 3'-end is of more importance for successful DNA amplification. As methylation seems to be more inhomogeneous at the 3'-end of the small promoter fragment it is even more likely for the U-primer to find a suitable template. MCA-Meth, in accordance with the sequencing data, also suggested an unmethylated promoter. However, the melting point peak position was slightly shifted towards a higher temperature; the curve itself seemed broadened and revealed a "shoulder". These changes in melting curve position and form may mirror the methylated state of the first 5–6 CpGs. While the position indicates a not completely unmethylated promoter, the width and shoulder of the curve suggests heterogeneity of promoter methylation in the sample. Thus, in MCA-Meth the extent of sequence methylation would determine the melting curve peak position while the heterogeneity of that sequence methylation would influence the melting curve appearance. The MCA-Meth result of U87MG illustrates the feasibility of the hypothesis that the extent of promoter methylation alters the melting curve peak position (Figure 3). Bisulfite sequencing of the fragment covered in the MCA-Meth analysis (CpGs -1 to 20 of the CpG island) revealed an inhomogeneous methylation pattern of that sequence. Accordingly the melting curve peak in MCA-Meth shifted to a position in between the hypermethylated and completely unmethylated controls (Figure 3). As both the 5'- and 3'-MSP-priming site were homogeneously methylated, MSP was significant for a methylated promoter. Thus in MCA-Meth a single shifted melting point position reflects the extent of sequence methylation and the shape of the melting curve indicates the heterogeneity of promoter methylation. Thus, should it be necessary to analyze the physiological relevance of the percentage of methylated CpG-sites in a given promoter or to define a threshold of methylated cytosines in a particular gene required for its silencing, MCA-Meth may provide a simple tool among the commonly used techniques to detect inhomogeneous methylation, besides bisulfite sequencing.

Our results encourage a back-to-back comparison of MCA-Meth and bisulfite sequencing in a large series of tumors harboring different numbers of methylated CpG dinucleotide residues in their sequence. This analysis will provide answers regarding the sensitivity and specificity of MCA-Meth in the detection of promoter methylation extent. It will also show the limits of this technique and will decide on its priority future use.

Another scenario is the co-detection of methylated and unmethylated alleles in the same sample represented by two distinct melting points. A co-detection of methylated and unmethylated alleles can be due to sample contamination with normal tissue or reflect single allelic promoter methylation. Here the respective peak heights if compared to a dilution series of methylated and unmethylated DNA may predict the prevailing methylation status. The data on *RASSF1A*-promoter methylation of glioma sample 7 and 30 may illustrate the significance of this distinction. Standard MSP co-detected methylated and unmethylated alleles in both samples. MSP failed to identify the prevailing methylation pattern. MCA-Meth, however, suggested the presence of less than 5% of methylated DNA for samples 7 compared to 72% of methylated DNA for sample 30 (Table 4). The prevailing methylation status was thus an unmethylated promoter for sample 7 and a methylated promoter for sample 30. Bisulfite sequencing supported these MCA-Meth data revealing an almost completely unmethylated promoter in sample 7 and a heavily methylated promoter in sample 30.

Of note, besides MCA-Meth there are several alternative techniques to reliably detect intermediate levels of promoter methylation, *i.e. *rtMSP, pyrosequencing and MALDI-TOF. However, in contrast to MCA-Meth these are more cost intensive and are in need for expensive hardware that is not easily accessible in some institutions. The all-in-one tube reaction of MCA-Meth avoiding a gel stage further adds to the advantages for MCA-Meth in screening or diagnostic settings.

We have described two methods that use bisulfite modified DNA and Melting Curve Analysis (MCA) to assess DNA methylation. Using the 5'-CpG island in the *RASSF1A*, *BLU *and *MGMT *gene promoters as a model system, we have demonstrated here that both MCA-MSP and MCA-Meth can be used to determine the methylation status in tumor samples and have discussed their advantages and possible shortcomings as compared to commonly used techniques for promoter methylation analyses.

## Conclusion

In our view, MCA-Meth combines the advantages of both MSP and bisulfite sequencing. Similar to MSP it is able to answer the question, whether a sample is hypermethylated or not. The advantage over the MSP is that it also addresses the question of the extent of promoter methylation, without going into the trouble of cloning, sequencing and analyzing each and every CpG, a major advantage over bisulfite sequencing. We do see however some restrictions to MCA-Meth in those diagnostic applications in which homogeneous priming-site-methylation reliably predicts gene silencing. Here, an heterogeneous sequence methylation status in between MSP primers may give ambiguous results in MCA-Meth and may need a second evaluation by standard MSP, MCA-MSP or bisulfite sequencing. Also, even so MCA-Meth provides data on the extent of promoter methylation it does not quantify the methylation status of individual CpG dinucleotides within the sequence. In these settings MCA-Meth may serve as a technically easy and almost ubiquitously available initial screening tool, followed up by more sophisticated quantitative techniques for selected candidates.

Those limitations apart, the advantages of MCA-MSP and MCA-Meth as outlined above prevail. Both may be used as feasible diagnostic tools for the majority of applications. MCA-Meth may in addition proof an alternative technique to fast screen for different levels of promoter hypermethylation.

## Abbreviations

DNA, deoxyribonucleic acid; m^5^C, 5-methylcytosine; PCR, polymerase chain reaction; MSP, methylation specific PCR; COBRA, combined bisulfite restriction analysis; Tm, melting temperature; MCA-MSP, Melting Curve Analysis-based Methylation Specific PCR; MCA-Meth, Melting Curve Analysis-based methylation assay; RASSF1A, Ras association domain family protein 1A; MGMT, 06-methyl-guanine-methyl-transferase; DMEM Medium, Dubelcco's modified Eagle's medium; RPMI Medium, Roswell Park Memorial Institute medium; FBS, fetal bovine serum; TE buffer, Tris-Cl EDTA buffer; dNTP, deoxynucleotide triphospate; DMSO, dimethyl sulfoxide; LB agar, Luria Bertani agar; X-Gal, 5-bromo-4-chloro-3-indolyl β-D-galactopyranoside; C_T_, thresold cycle; IMD, in vitro methylated DNA.

## Competing interests

The author(s) declare that they have no competing interests.

## Authors' contributions

AL and EU carried out the MCA-MSP and MCA-Meth studies, and PL performed the MSP experiments under the supervision of JSC. AL also performed the bisulfite sequencing assays under the supervision of WM and participated in the study design and drafting of the manuscript. JSC, AvD and WM conceived of the study, participated in its design, coordination and in the draft of the manuscript. All authors read and approved the final manuscript.

## Pre-publication history

The pre-publication history for this paper can be accessed here:


